# Cardiovascular autonomic regulation, inflammation and pain in rheumatoid arthritis

**DOI:** 10.1016/j.autneu.2017.09.003

**Published:** 2017-12

**Authors:** Ahmed M. Adlan, Jet J.C.S. Veldhuijzen van Zanten, Gregory Y.H. Lip, Julian F.R. Paton, George D. Kitas, James P. Fisher

**Affiliations:** a College of Life and Environmental Sciences, University of Birmingham, Edgbaston, Birmingham B15 2TT, UK; b University of Birmingham Centre of Cardiovascular Sciences, City Hospital, Birmingham B18 7QH, UK; c School of Physiology, Pharmacology & Neuroscience, Biomedical Sciences, University of Bristol, Bristol BS8 1TD, UK; d Department of Rheumatology, Dudley Group NHS Foundation Trust, Russells Hall Hospital, Dudley, West Midlands DY1 2HQ, UK

**Keywords:** ANOVA, analysis of variance, BP, blood pressure, BMI, body mass index, CI, confidence interval, CPT, cold pressor test, DAS, disease activity score, ECG, electrocardiogram, EDR, ECG-derived respiration, FVC, forearm vascular conductance, HF, high frequency, HR, heart rate, HRV, heart rate variability, hs-CRP, high sensitivity C-reactive protein, HTN, hypertensive, IL, interleukin, IRR, adjusted incidence ratio, LF, low frequency, LSD, least significant difference, LVC, leg vascular conductance, NC, normotensive control, PASAT, paced auditory serial addition test, pNN50%, proportion of RR intervals differing by > 50 ms from previous RR interval, RA, rheumatoid arthritis, RMSSD, square root of the mean of the sum of successive differences, SD, standard deviation, SDNN, standard deviation of all RR [NN] intervals, SPSS, statistical analysis software package, TNF, tumour necrosis factor, TP, total power, VAS, visual analogue scale, Autonomic nervous system, Stress, Physiological, Parasympathetic

## Abstract

**Background:**

Rheumatoid arthritis (RA) is a chronic inflammatory condition characterised by reduced heart rate variability (HRV) of unknown cause. We tested the hypothesis that low HRV, indicative of cardiac autonomic cardiovascular dysfunction, was associated with systemic inflammation and pain. Given the high prevalence of hypertension (HTN) in RA, a condition itself associated with low HRV, we also assessed whether the presence of hypertension further reduced HRV in RA.

**Methods:**

In RA-normotensive (*n* = 13), RA-HTN (*n* = 17), normotensive controls (NC; *n* = 17) and HTN (*n* = 16) controls, blood pressure and heart rate were recorded. Time and frequency domain measures of HRV along with serological markers of inflammation (high sensitivity C-reactive protein [hs-CRP], tumour necrosis factor-α [TNF-α] and interleukins [IL]) were determined. Reported pain was assessed using a visual analogue scale.

**Results:**

Time (rMSSD, pNN50%) and frequency (high frequency power, low frequency power, total power) domain measures of HRV were lower in the RA, RA-HTN and HTN groups, compared to NC (*p* = 0.001). However, no significant differences in HRV were noted between the RA, RA-HTN and HTN groups. Inverse associations were found between time and frequency measures of HRV and inflammatory cytokines (IL-6 and IL-10), but were not independent after multivariable analysis. hs-CRP and pain were independently and inversely associated with time domain (rMMSD, pNN50%) parameters of HRV.

**Conclusions:**

These findings suggest that lower HRV is associated with increased inflammation and independently associated with increased reported pain, but not compounded by the presence of HTN in patients with RA.

## Highlights

• Rheumatoid arthritis (RA) is a chronic inflammatory condition accompanied by low heart rate variability (HRV).
• Important autonomic-immune interactions are suggested, but have not been thoroughly examined in RA.
• We show that low HRV in RA is associated with increased serum inflammatory cytokine levels and patient-reported pain.
• In our patients with RA, reductions in HRV were not compounded by the presence of hypertension.

## 1 Introduction

Rheumatoid arthritis (RA) is a chronic inflammatory condition associated with substantially increased cardiovascular mortality and risk (Solomon et al., 2003, Pujades-Rodriguez et al., 2016). In a large epidemiological study RA was associated with increased risk of myocardial infarction (adjusted incidence ratio [IRR] = 1.43, 95% confidence interval [CI] 1.21–1.70), heart failure (IRR = 1.61,1.43–1.83), cardiac arrest (IRR = 2.26, 1.69–3.02) and unheralded coronary death (IRR = 1.60, 1.18–2.18) (Pujades-Rodriguez et al., 2016). Low heart rate variability (HRV) indicative of reduced cardiac parasympathetic function predicts mortality risk following myocardial infarction (Bigger et al., 1992, La Rovere et al., 1998) and hence may contribute to the increased cardiovascular risk seen in RA. Studies to date have shown that HRV is reduced in RA, compared to healthy controls (Adlan et al., 2014), although the mechanisms are not known.

Animal studies have identified direct and reciprocal relationships between parasympathetic activity and inflammatory cytokines (Borovikova et al., 2000, Bernik et al., 2002, Fairchild et al., 2009). Intra-peritoneal administration of the pro-inflammatory cytokine tumour necrosis factor-alpha (TNF-α) in mice, reduced a HRV derived index of parasympathetic activity (Fairchild et al., 2009) while pharmacological (Borovikova et al., 2000, Bernik et al., 2002) and electrical (Borovikova et al., 2000) stimulation of the vagus nerve attenuates the release of inflammatory cytokines. In healthy humans acute inflammation (precipitated by an influenza vaccine) attenuated heart rate recovery following exercise (marker of parasympathetic activity) (Jae et al., 2010). However, studies of RA patients that have examined the associations between inflammation and cardiac parasympathetic activity have been limited (e.g., cytokine concentrations not assessed) and reported equivocal results (Adlan et al., 2014). Another possible explanation for the observed reduction in HRV in RA patients is increased patient-reported pain. Central pain pathways are known to overlap with areas of autonomic control (e.g., nucleus of the solitary tract) (Benarroch, 2006) and in a recent meta-analysis HRV was found to be lower in patients with chronic pain (Tracy et al., 2016). Despite this, the associations between pain and cardiac autonomic function in RA remain unknown. Furthermore, given the high prevalence of hypertension in RA (Panoulas et al., 2007), and that HRV is reduced in hypertension (Singh et al., 1998), it remains to be proven/seen whether the presence of hypertension in RA exacerbates the reductions in HRV. Vasoconstrictor sympathetic nerve activity is elevated in RA patients and associated with pain and inflammation (Adlan et al., 2017). In the absence of direct intraneural recordings of cardiac autonomic activity in humans, HRV analyses have provided a useful indirect surrogate. However, the target-organ specific control of pre-motor and motor neurones (Polson et al., 2007, Simms et al., 2007) along with local modulation of receptor signalling, means that observations from one region (e.g., peripheral vasculature) cannot be generalised to another (e.g., heart). Therefore, important questions remain regarding the consequences of RA to cardiac autonomic regulation as assessed with HRV, and the underlying mechanisms.

The autonomic nervous system plays a key role in orchestrating the cardiovascular response to stressors (Dampney, 1994, Wehrwein and Joyner, 2013). Cardiovascular responses to mental stress (Matthews et al., 2004) or a cold pressor test (CPT; immersion of a limb into cold water) (Treiber et al., 2003) can predict the development of cardiovascular disease. Impaired cardiovascular responses to stressors have been demonstrated in the majority of prior studies in RA patients (e.g., orthostasis, deep-breathing, Valsalva manoeuvre and handgrip) (Adlan et al., 2014). The diastolic blood pressure response to CPT in RA patients has been examined in one study and were reported as being attenuated (Bidikar and Ichaporia, 2010), while the cardiovascular responses to mental stress have been conflicting (Geenen et al., 1996, Veldhuijzen van Zanten et al., 2005, Motivala et al., 2008, Veldhuijzen van Zanten et al., 2008). These conflicting results may reflect opposing effects of inflammatory cytokines on vascular resistance responses to mental stress. Inflammatory cytokines have vasodilatory actions (Takizawa et al., 1997, Clapp et al., 2005), but may also exaggerate vasoconstrictor pathways (Wassmann et al., 2004, Veldhuijzen van Zanten et al., 2008). The vascular responses to mental stress are also regionally differentiated (Folkow et al., 1964), but it is not known how the arm and leg vascular responses to mental stress are affected by RA, and if these responses are related to inflammatory cytokine concentration or patient-reported pain.

In this observational, case-control study of patients with RA and matched-control participants we determined how HRV and cardiovascular responses to CPT and mental stress (paced auditory serial addition test; PASAT) were associated with pain (visual analogue scale, VAS) and baseline serum inflammatory cytokine concentrations. We hypothesised that HRV derived indices of cardiac parasympathetic would be attenuated and cardiovascular reactivity would be greater in individuals with increased inflammatory cytokine concentrations and more reported pain. We further hypothesised that the presence of hypertension in RA would exacerbate the cardiovascular autonomic alterations.

## 2 Materials and methods

### 2.1 Participants

The study was approved by the National Research and Ethics Service Committee West Midlands - Edgbaston (11/WM/0298). Written informed consent was obtained from all participants, in accordance with the Declaration of Helsinki (2013). A total of sixty-six participants were recruited, the general and clinical characteristics of which are provided in a previous study testing other hypotheses (Adlan et al., 2017). Thirty patients with a diagnosis of RA (based on the 1987 American College of Rheumatology criteria (Arnett et al., 1988)) were recruited from the rheumatology clinics at Russells Hall Hospital, Dudley, UK and Sandwell General Hospital, West Bromwich, UK including normotensive (RA *n* = 13, mean age ± SD 56 ± 12 yr, 8 women, body mass index [BMI] geometric mean 28, 95% confidence interval 25–30 kg/m^2^) and hypertensive (RA-HTN *n* = 17, age 61 ± 10 yr, 12 women, BMI 30, 26–33 kg/m^2^). Thirty-three normotensive and hypertensive control participants of a similar age and BMI were recruited from the hospitals and surrounding areas (NC *n* = 17, age 54 ± 13 yr, 10 women, BMI 26, 24–29 kg/m^2^; HTN *n* = 16, age 60 ± 10, 11 women, BMI 26, 25–27 kg/m^2^). Exclusion criteria included: age < 18 or > 75 years; atrial fibrillation or other heart rhythm disorder, significant valvular disease, coronary artery disease, diabetes, ischemic stroke, chronic renal failure, liver impairment, hormone replacement therapy and those who are pregnant or who might be pregnant. NC participants were free from major illnesses, whilst HTN participants either had a prior diagnosis of hypertension or BP ≥ 140/90 mm Hg.

### 2.2 Experimental protocol

Following an overnight fast (from food, caffeine and alcohol), participants attended the research laboratory at 09:00 h. Medications were withheld on the morning of testing. A detailed clinical history was taken and physical examination performed in RA patients to count the number of swollen and tender joints in order to determine the disease activity score (DAS28-CRP) (Wells et al., 2009). A visual analogue scale (VAS) was used as a measure of pain (Huskisson, 1974). Height and weight was measured, and BMI was determined (weight/height^2^). Subsequent measurements were performed in a temperature-controlled room under uniform conditions with participants resting quietly in the supine position.

### 2.3 Measurements

HR was continuously recorded using a lead II ECG (BioAmp, ADInstruments, Bella Vista, Australia). Beat-to-beat BP was recorded using finger photoplethysmography (Portapres, Finapres Medical Systems, Amsterdam, The Netherlands) and was calibrated with brachial BP recordings using an automated sphygmomanometer (Omron 705IT, Omron Corporation, Hoopddorp, The Netherlands). Leg blood flow (venous occlusion strain gauge plethysmography, Hokanson EC-6 plethysmograph, D E Hokanson, Bellevue, United States of America, USA) (Joyner et al., 2001) was recorded during rest, test and recovery phases of the CPT and PASAT, as described in detail elsewhere (Adlan et al., 2017). During the PASAT, forearm blood flow was also recorded. Leg and forearm vascular conductance (LVC, FVC) were calculated as Blood flow (ml/100 ml/min)/Mean BP (mm Hg) × 1000. Blood samples for inflammatory markers were centrifuged immediately and plasma stored at − 80 °C. Commercially available ELISA kits were used to determine hs-CRP (MP Biomedicals, California, USA) and cytokines (IL-6, TNF-α, IL-10; BioSupply UK, Bradford, UK).

### 2.4 HRV

In accordance with guidelines from the Task Force of the European Society of Cardiology and the North American Society of Pacing Electrophysiology (TaskForce, 1996) time domain, frequency domain (fast Fourier transform) and non-linear (SD1 and SD2 standard deviations of the Poincare plot) indices of short-term HRV were determined from a 10 min resting period (Kubios HRV, Kuopio, Finland). Data was pre-screened for ectopics and these were corrected using the Kubios software (accounted for < 1% of all recordings) (TaskForce, 1996). HRV indices of cardiac parasympathetic activity included RMSSD (square root of the mean of the sum of successive differences), pNN50% (proportion of RR intervals differing by > 50 ms from previous RR interval) and high frequency power spectral density (HF, 0.15–0.4 Hz). Power spectral density at the low frequency range (LF, 0.04–0.15 Hz) was used as a combined index of cardiac sympathetic and parasympathetic activity. The LF/HF ratio has been used as an estimate of ‘sympathovagal balance’, however this concept has been debated (Parati et al., 2006, Taylor and Studinger, 2006). Indices of total HRV included SDNN (standard deviation of all RR [NN] intervals) and total power (TP range, 0–0.5 Hz). SD1 provides an estimate for short term HRV whilst SD2 is representative of long term HRV (Woo et al., 1994) and is influenced by both parasympathetic and sympathetic activity (Mourot et al., 2004). The detrended fluctuation analysis short-term coefficient (DFA-α1) was included in light of suggested utility in quantifying short-term changes in HRV due to autonomic activation, but relative insensitivity to respiratory rate (Sassi et al., 2015). Estimates of the respiratory rate (ECG-derived respiration, EDR) were also obtained (Kubios HRV).

### 2.5 Cardiovascular reactivity

The CPT and PASAT were preceded by 4-minute resting baseline and followed by 4-minute recovery measurements. During the CPT the right hand was immersed completely in a container of cold water at 4°C for 2 min. During the PASAT stress test a series of single digit numbers were presented to the participants for 6 min using a pre-recorded audio file on a computer. Participants were instructed to add each number they heard to the previous number presented to them, and retain the last number to add to the next number they heard (Veldhuijzen van Zanten et al., 2005). In order to make the task progressively more challenging the numbers were presented every 3.5 s, 3.0 s and 2.5 s respectively, in three consecutive blocks each lasting 2 min. An experimenter checked their responses against the correct answers and alerted the participant with a loud buzzer noise with each incorrect answer, hesitation or once during every 10 additions if no mistakes were made. Finally, in order to increase social evaluation participants were instructed to view themselves in a mirror for the duration of the mental stress test. Pain and stress ratings (10-point scale) were taken after the CPT and PASAT, respectively.

### 2.6 Data and statistical analysis

Data was acquired using the Powerlab 16/35 data acquisition system and a personal computer equipped with LabChart Pro software (ADInstruments, Bella Vista, Australia). Cardiovascular variables were sampled at 1 kHz, and beat-to-beat values of HR, systolic BP, diastolic BP and mean BP calculated. Cardiovascular variables were averaged during rest, test and recovery phases to provide absolute values. Differences between baseline, test and recovery phases of CPT and PASAT were reported as absolute change. Some participants declined or were unable to complete the cardiovascular reactivity tests, thus these were omitted from the analyses and participant numbers are stated in the legend of each Table and Figure. BP, HR, forearm and leg blood flow and FVC, LVC were averaged during rest, test and recovery phases, and change from rest was calculated.

Statistical analysis was performed using SPSS software, version 19 (SPSS Inc., Chicago, Ilinois). Continuous variables were tested for normality using the Shapiro-Wilk test. Non-normally distributed data were logarithmically transformed and the distribution re-checked with a Shapiro Wilk test. Data that were normally distributed were then assessed using an ANOVA (least significant difference [LSD] post-hoc) for continuous variables, while data that were still not normally distributed were analysed with a Kruskal Wallis test (using original, non-log transformed data). For cardiovascular reactivity, an ANOVA with repeated measures (Bonferroni adjustments for multiple comparisons) was used to test for significant differences between groups and phase (rest, test, recovery) during CPT and PASAT. Post-hoc LSD analysis was performed if significant group x phase interactions were found. Group differences in changes from baseline (∆ test, ∆ recovery) in HR, BP, leg and forearm blood flow, LVC and FVC were tested using a one-way ANOVA. Associations between autonomic parameters and inflammation were assessed before (Pearson product/Spearman's rank correlation coefficient) and after adjustment for potential confounders (including age, sex, BMI, presence of hypertension, RA diagnosis and haemoglobin concentration) using regression analyses. Normally distributed data are expressed as mean ± SD and non-normally distributed data are displayed as geometric mean (95% CI); and frequency (%) for categorical variables. A *p* value of < 0.05 was considered statistically significant.

## 3 Results

Resting HR was similar in RA and RA-HTN groups but higher compared to NC and HTN controls (*p* = 0.008, Table 1). BP was similar in RA-HTN and HTN groups but higher than RA and NC (*p* < 0.001). Leg blood flow was higher in RA and RA-HTN groups compared to NC (*p* = 0.047) and similar to HTN controls. There were no significant differences in resting LVC (*p* = 0.148), forearm blood flow (*p* = 0.541) or FVC (*p* = 0. 782) between the groups.

**Table 1:** Haemodynamic and heart rate variability data.

	RA	RA-HTN	NC	HTN	P value
N	13	17	17	16	
HR, bpm	66 ± 10*†	65 ± 10*	57 ± 7	60 ± 7	0.008
Systolic BP, mm Hg	128 (123–135)†‡	153 (145–161)*	123 (118–128)	146 (134–158)*	< 0.001
Diastolic BP, mm Hg	79 ± 6‡	87 ± 10*	75 ± 6	84 ± 11*	< 0.001
Mean BP, mm Hg	95 (91–100)†‡	109 (104–114)*	89 (82–95)	105 (98–112)*	< 0.001
Leg blood flow, ml/100 ml/min	2.0 (1.5–2.6)*	2.0 (1.4–2.8)*	1.2 (0.9–1.7)	1.4 (1.0–1.8)	0.047
LVC, units	21 (15–27)	18 (12–26)	14 (10–19)	13 (10–17)	0.148
Forearm blood flow, ml/100 ml/min^a^	2.8 (1.9–4.1)	3.5 (2.2–5.5)	2.5 (1.9–3.3)	2.4 (1.9–3.0)	0.541
FVC, units^a^	29 (20–42)	31 (18–51)	27 (21–36)	23 (17–30)	0.782
rMSSD, ms	25 (18–36)*	20 (13–29) *	48 (38–60)	29 (20–40)	0.003
pNN50, %	5 (2 − 12)*	3 (1–7)*	18 (11–29)	5 (2–12)*	0.005
SD1	18 (13–26)*	14 (9–21)*	38 (27–43)	20 (14–29)	0.003
SD2	48 (36–63)*	46 (35–61)*	79 (67–94)	58 (45–76)	0.005
DFA-α1	1.0 (0.9–1.2)	1.2 (1.0–1.4)	1.0 (0.9–1.3)	1.1 (1.0–1.3)	0.136
HF, ms^2^	256 (131–500)*	116 (52–260)*	759 (436–1322)	240 (116–496)*	< 0.001
LF, ms^2^	272 (153–482)*	245 (132–456)*	1097 (746–1612)	357 (186–683)*	0.001
VLF, ms^2^	556 (304–1020)*	567 (315–1017)*	1431 (951–2151)	946 (552–1621)	0.025
TP, ms^2^	1135 (631–2042)*	1014 (569–1807)*	3104 (2010–4793)	1665 (943–4793)	0.010
HF, nu	49 ± 15‡	34 ± 14	42 ± 15	41 ± 15	0.053
LF, nu	51 ± 15‡	67 ± 14	58 ± 15	59 ± 15	0.053
LF/HF ratio	1.1 (0.7–1.5)‡	2.1 (1.5–3.0)	1.5 (1.0–2.1)	1.5 (1.0–2.1)	0.049
EDR, Hz	0.218 ± 0.032	0.222 ± 0.050	0.202 ± 0.051	0.232 ± 0.047	0.336

Normally distributed data are expressed as mean ± standard deviation. Non-normally distributed data are displayed as geometric mean (95% confidence intervals). Statistical differences were tested using a one-way ANOVA with post hoc LSD or Kruskal Wallis with post hoc Dunn-Bonferroni.

Significance *p* ≤ 0.05. Post hoc *p* ≤ 0.05 * v NC, † v HTN, ‡v RA-HTN.

BMI = body mass index, BP = blood pressure, DFA = detrended fluctuation analysis, EDR, ECG derived respiration, HF = high frequency power (0.15–0.4 Hz), HR = heart rate, LF = low frequency power (0.04–0.15 Hz), RA = rheumatoid arthritis, pNN50 = NN50 as a percentage of all NN intervals, rMSSD = root mean square of successive differences, TP, total power (0.04–0.5 Hz), VLF = very low frequency power (0–0.04 Hz).

a Forearm blood flow and FVC were determined during the rest period of the PASAT. RA *n* = 10, RA-HTN n = 10, NC *n* = 16, HTN *n* = 14.

### 3.1 HRV

Time domain (rMSSD, pNN50%), frequency domain (HF, LF) and non-linear (SD1, SD2) parameters of HRV were similar in RA, RA-HTN and HTN groups and lower compared to NC (Table 1) (HTN vs. NC, *p* = 0.092). Very low frequency (VLF) and TP were also lower in RA and RA-HTN groups compared to NC. RA normotensive patients had higher normalised HF power (*p* = 0.053), but lower normalised LF power (p = 0.053) and LF/HF ratio (*p* < 0.05) compared to RA-HTN. Time domain (rMSSD and pNN50), frequency domain (TP, LF power, HF power) and non-linear (SD1, SD2) parameters of HRV were inversely associated with hs-CRP (Table S1). Ln (hs-CRP) was independently associated with rMSSD, pNN50, LF power, HF power, SD1, SD2, following adjustments for multiple variables (i.e., age, sex, BMI, presence of hypertension, RA diagnosis and serum haemoglobin concentration) (Table 2). Inflammatory cytokines were inversely associated with HRV parameters (IL-6 and rMSSD, LF power, SD1, SD2; IL-10 and LF/HF ratio; trend for TNF-α and LF power) although these associations were no longer present after multivariable analysis. Pain was independently and inversely associated with time domain (rMSSD, pNN50) and non-linear (SD1, SD2) parameters of HRV. LF power and HF power HRV indices were inversely associated with pain, although attenuated following multivariable adjustment. EDR was not different between groups (Table 1), and re-analysis performed after omitting six participants (3 NC and 3 RA) with a respiratory frequency > 0.15 Hz provided similar results (data not shown).

**Table 2:** Association between inflammation, pain and heart rate variability before and after multivariable adjustment.

	N	Univariable^a^		Multivariable^b^		
		Rho	P	R^2^	F	P
Dependent variable: rMSSD						
hs-CRP	57	− 0.420	0.001	0.334	3.036	0.088
Ln (hs-CRP)				0. 490	10.481	0.002⁎
IL-6	62	− 0.258	0.043	0.216	0.230	0.633
Ln (IL-6 + 1)				0.307	0.309	0.581
VAS	63	− 0.437	< 0.001	0.303	7.015	0.011⁎
Ln (VAS + 1)				0.384	7.045	0.010⁎

Dependent variable: pNN50						
hs-CRP	57	− 0.430	0.001	0.388	3.270	0.077
Ln (hs-CRP)				0.458	10.060	0.003
VAS	63	− 0.419	0.001	0.356	7.179	0.010⁎
Ln (VAS + 1)				0.526	7.364	0.009⁎

Dependent variable: LF power						
hs-CRP	57	− 0.371	0.004	0.210	1.703	0.198
Ln (hs-CRP)				0.356	5.408	0.024⁎
VAS	63	− 0.367	0.003	0.167	2.496	0.120
Ln (VAS + 1)				0.283	4.105	0.048⁎
IL-6	62	− 0.270	0.034	0.130	0.093	0.761
Ln (IL-6 + 1)				0.230	0.099	0.755

Dependent variable: HF power						
hs-CRP	57	− 0.348	0.008	0.311	1.417	0.240
Ln (hs-CRP)				0.446	5.848	0.019⁎
VAS	63	− 0.371	0.003	0.209	1.928	0.171
Ln (VAS + 1)				0.313	3.043	0.087

Dependent variable: LF/HF ratio						
IL-10	62	− 0.262	0.040	− 0.01	2.639	0.110
Ln (IL-10 + 1)				0.125	3.891	0.054

Dependent variable: SD1						
hs-CRP	57	− 0.420	0.001	0.334	3.038	0.088
Ln (hs-CRP)				0.490	10.484	0.002⁎
IL-6	62	− 0.258	0.043	0.216	0.230	0.633
Ln (IL-6 + 1)				0.307	0.309	0.581
VAS	63	− 0.437	< 0.001	0.303	7.017	0.011⁎
Ln (VAS + 1)				0.384	7.046	0.010⁎

Dependent variable: SD2						
hs-CRP	57	− 0.344	0.009	0.236	2.341	0.132
Ln (hs-CRP)				0.370	5.462	0.024⁎
IL-6	62	− 0.313	0.013	0.164	0.125	0.725
Ln (IL-6 + 1)				0.259	0.095	0.760
VAS	63	− 0.390	0.002	0.227	4.531	0.038⁎
Ln (VAS + 1)				0.317	4.625	0.036⁎

a Spearman's rank.

b After adjustment for age, sex, BMI, presence of hypertension, RA diagnosis and haemoglobin concentration.

⁎ p < 0.05

### 3.2 Cardiovascular reactivity

**Fig. 1:**
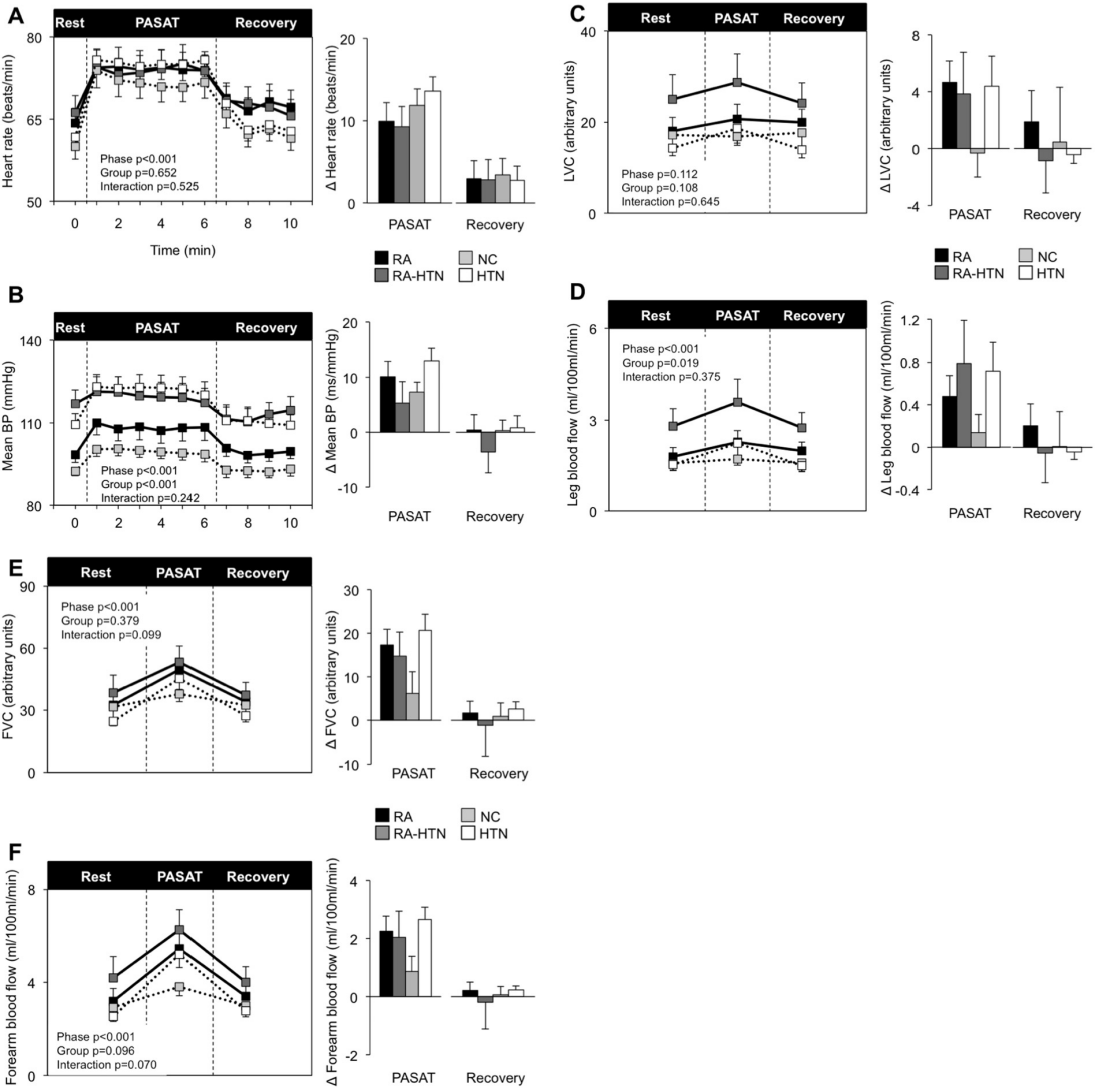
Cardiovascular reactivity to the PASAT mental stress task. Heart rate (Panel A), mean blood pressure (Panel B), leg vascular conductance (Panel C), leg blood flow (Panel D), forearm vascular conductance (Panel C), forearm blood flow (Panel D) during rest, mental stress test (PASAT) and recovery. Data represented as group means ± SEM. Times series is shown on the left. Significance for phase (rest, PASAT and recovery), group (RA, RA-HTN, NC and HTN) and interaction were assessed using ANOVA with repeated measures. Bar charts on the right represent changes from baseline. Significant group differences were assessed using a one-way ANOVA. *p ≤ 0.05. RA *n* = 10, RA-HTN *n* = 10, NC *n* = 16, HTN *n* = 14. For leg blood flow and leg vascular conductance RA-HTN *n* = 9. BP = blood pressure, CPT = cold pressor test, FVC = forearm vascular conductance, HTN = hypertensive, LVC = leg vascular conductance, NC = normotensive control, PASAT = paced auditory serial arithmetic task, RA = rheumatoid arthritis.

As expected HR, BP, leg blood flow, forearm blood flow and FVC rose during the PASAT in all groups. There were no significant differences in HR, BP, LVC or leg blood flow responses to PASAT between the groups (Fig. 1). No difference in self-reported stress was found between groups (4.9 ± 3.7, 5.3 ± 3.7, 5.3 ± 3.3, 4.4 ± 2.7 max score 10; *p* = 0.962). ∆ Systolic BP PASAT was inversely associated with IL-6 (Table S2), while there were trends for positive association between leg vascular responses to PASAT and inflammation (hs-CRP, IL-6). Following multivariable analysis IL-10 and Ln (IL-10) were independently positively associated with ∆ HR PASAT (*p* = 0.020 and 0.029), ∆ FVC PASAT (*p* = 0.039 and 0.048), while ∆ mean BP PASAT was independently associated with the number of tender joints (*p* = 0.029, Table 3).

**Table 3:** Association between inflammation, pain and mental stress responses before and after multivariable adjustment.

	N	Univariable^a^		Multivariable^b^		
		Rho	P	R^2^	F	P
Dependent variable: ∆ HR PASAT						
IL-10	50	0.187	0.194	0.203	5.831	0.020⁎
Ln (IL-10 + 1)				0.191	5.107	0.029⁎
VAS	50	− 0.244	0.088	0.119	1.257	0.269
Ln (VAS + 1)				0.103	0.493	0.487

Dependent variable: ∆ Mean BP PASAT						
Number of tender joints	20	− 0.376	0.102	0.610	5.996	0.029⁎

Dependent variable: ∆ Systolic BP PASAT						
Number of tender joints	20	− 0.403	0.078	0.529	4.455	0.055

Dependent variable: ∆ leg blood flow						
hs-CRP	45	0.287	0.056	0.133	0.451	0.506
Ln (hs-CRP)				0.152	1.297	0.262

Dependent variable: ∆ LVC						
hs-CRP	45	0.246	0.103	0.083	0.337	0.565
Ln (hs-CRP)				0.112	1.580	0.217
IL-6	49	0.237	0.101	0.085	0.473	0.495
Ln (IL-6 + 1)				0.108	1.545	0.221

Dependent variable: ∆ forearm blood flow						
IL-10	50	0.198	0.168	0.220	5.435	0.025⁎
Ln (IL-10 + 1)				0.188	3.563	0.066
DAS28-CRP^c^	20	− 0.394	0.086	0.434	2.008	0.182
Tender	20	− 0.394	0.110	0.407	3.253	0.095

Dependent variable: ∆ FVC						
IL-10	50	0.250	0.080	0.197	4.555	0.039⁎
Ln (IL-10 + 1)				0.190	4.151	0.048⁎
DAS28-CRP^c^	20	− 0.402	0.079	0.361	1.627	0.226

a Spearman's rank.

b After adjustment for age, sex, BMI, presence of hypertension, RA diagnosis and haemoglobin concentration.

c After adjustment for age, sex, BMI, presence of hypertension, haemoglobin concentration and RA duration.

⁎ p < 0.05.

**Fig. 2:**
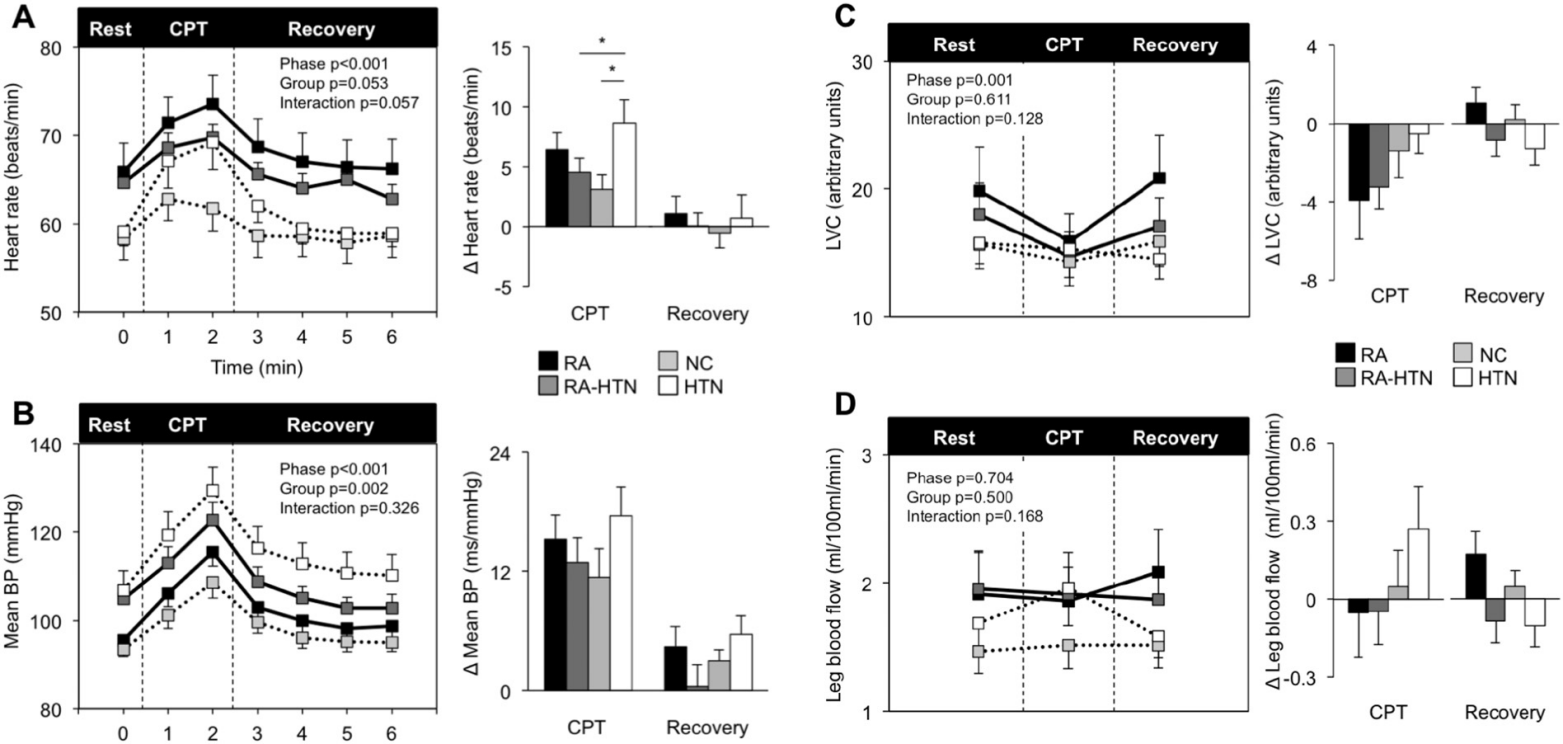
Cardiovascular reactivity to the cold pressor test. Heart rate (Panel A), mean blood pressure (Panel B), leg vascular conductance (LVC) (Panel C) and leg blood flow (Panel D) during rest, cold pressor test (CPT) and recovery. Data represented as group means ± SEM. Times series is shown on the left. Significance for phase (rest, CPT and recovery), group (RA, RA-HTN, NC and HTN) and interaction were assessed using ANOVA with repeated measures. Bar charts on the right represent changes from baseline. Significant group differences were assessed using a one-way ANOVA. *p ≤ 0.05. RA *n* = 12, RA-HTN *n* = 16, NC *n* = 16, HTN *n* = 16. For leg blood flow and leg vascular conductance RA-HTN *n* = 15, HTN *n* = 15. BP = blood pressure, CPT = cold pressor test, HTN = hypertensive, LVC = leg vascular conductance, NC = normotensive control, RA = rheumatoid arthritis.

During the CPT, HR and BP increased while LVC was reduced and leg blood flow unchanged (Fig. 2). The HTN group had a significantly greater rise in HR compared to NC and RA-HTN (*p* = 0.049). There were no statistically significant differences in BP or LVC responses between the groups. RA, RA-HTN and HTN patients tended to have higher pain rating (RA geometric mean 8.3, 95% CI 6.1–9.5; RA-HTN 7.8, 6.1–9.5; NC 5.6, 4.3–7.2; 7.4, HTN 7.4, 5.4–8.6 max score 10; *p* = 0.10) compared to NC. ∆ HR CPT was positively and independently associated with the inflammatory cytokines TNF-α and IL-10 (Table S2; Table 4).

**Table 4:** Association between inflammation, pain and cold pressor test responses before and after multivariable adjustment.

	N	Univariable^a^		Multivariable^b^		
		Rho	P	R^2^	F	P
Dependent variable: ∆ HR CPT						
TNF-α	60	0.254	0.050	0.175	4.715	0.034⁎
Ln (TNF-α + 1)				0.199	6.392	0.015⁎
IL-10	60	0.299	0.020	0.196	6.192	0.016⁎
Ln (IL-10 + 1)				0.199	6.425	0.014⁎
DAS28-CRP^c^	28	− 0.308	0.111	0.399	0.901	0.354

Dependent variable: ∆ Mean BP CPT						
TNF-α	60	0.232	0.074	0.094	0.724	0.399
Ln (TNF-α + 1)				0.118	2.160	0.148
IL-10	60	− 0.179	0.171	0.143	3.783	0.057
Ln (IL-10 + 1)				0.119	2.223	0.142

Dependent variable: ∆ Systolic BP CPT						
IL-10	60	0.112	0.395	0.133	3.396	0.071
Ln (IL-10 + 1)				0.089	0.701	0.406

Dependent variable: ∆ Diastolic BP CPT						
TNF-α	60	0.245	0.059	0.077	1.664	0.203
Ln (TNF-α + 1)				0.105	3.339	0.073
IL-10	60	0.212	0.103	0.109	3.536	0.066
Ln (IL-10 + 1)				0.101	3.086	0.085

a Spearman's rank.

b After adjustment for age, sex, BMI, presence of hypertension, RA diagnosis and haemoglobin concentration.

c After adjustment for age, sex, BMI, presence of hypertension, haemoglobin concentration and RA duration.

⁎ p < 0.05.

## 4 Discussion

In this study, we observed a reduction in time and frequency domain measures of HRV in patients with RA, and show for the first time that inverse associations exist between HRV and inflammation (hs-CRP, IL-6), with the association between Ln (hs-CRP) and HRV persisting after adjustment for potential confounders (e.g., age, sex). Notably, HRV (rMMSD, pNN50%) was independently and inversely associated with reported pain, but the presence of HTN in RA did not compound the reduction in HRV.

The underlying mechanisms for reduced HRV observed in RA have hitherto remained obscure. Cytokines may reduce HRV via afferent pathways, efferent pathways, or central sites of integration. Intra-peritoneal administration of the pro-inflammatory cytokine TNF-α in mice, reduced a parasympathetic index of HRV (i.e., SDNN) (Fairchild et al., 2009). Furthermore, direct administration of IL-6 into the nucleus of the solitary tract (a key autonomic cardiovascular regulatory site) reduced baroreflex sensitivity in rats (Takagishi et al., 2010). We have previously identified that the cardiac baroreflex sensitivity is reduced in RA (Adlan et al., 2017). In the present study, we observed a weak inverse association between HRV indices and serological markers of inflammation (hs-CRP, IL-6, TNF-α), and a significant independent association between Ln (hs-CRP) and HRV after adjustment for potential confounders (e.g., age, sex, BMI). hs-CRP, an acute phase reactant and non-specific inflammatory marker that predicts cardiovascular mortality in healthy humans (Kuller et al., 1996, Ridker et al., 1997), has been shown to be inversely associated with HRV in healthy humans (Aeschbacher et al., 2017). The relatively day-to-day stability of hs-CRP, compared to other cytokines (IL-6, TNFα), may explain why it is the inflammatory marker that demonstrates the most robust association with HRV.

A strong independent and inverse relationship was found between reported pain and HRV. These findings are broadly in agreement with studies showing that HRV is reduced in chronic pain conditions, such as fibromyalgia (Tracy et al., 2015). Intriguingly, in patients with fibromyalgia, resistance exercise training related increases in HRV were correlated with reductions in pain (Figueroa et al., 2008). Furthermore, experimentally induced pain in healthy individuals causes a reduction in HRV consistent with a fall in cardiac parasympathetic activity (Koenig et al., 2014) and increased sympathetic nerve activity (Bruehl and Chung, 2004). Indeed, intramuscular infusion of hypertonic saline increases muscle sympathetic nerve activity in some individuals but decreases in others during an hour of muscle pain, yet that there are no differences in HRV between groups (Kobuch et al., 2015). A complex functional interaction exists between neural structures implicated in the regulation of the autonomic nervous system and the sensation of pain within the central and peripheral nervous systems (Benarroch, 2006). Convergent inputs from nociceptors and viscerosensory receptors are received by multiple brain regions that are highly interconnected with central autonomic regulatory sites (e.g., insula, amygdala, parabrachial nucleus, nucleus of the solitary tract, ventrolateral reticular formation) and choreograph an autonomic cardiovascular response upon stimulation (Paton et al., 2005). Inflammatory cytokines can modulate pain perception, and in RA patients central nociceptive activity and limbic system activation have been shown to be acutely blocked by TNF-α inhibition (Hess et al., 2011).

Given the widespread prevalence of HTN in RA, the inclusion of separate RA groups with and without HTN, and a HTN group without RA, is a strength of our study. Cardiac parasympathetic regulation (i.e., HRV and cardiovagal baroreflex sensitivity) is reportedly reduced in HTN patients (Singh et al., 1998) and independently predict all-cause mortality in this condition (Ormezzano et al., 2008). It is possible that the concomitant presence of RA and HTN would compound the reduction in HRV, however we observed no HRV differences between those RA patients with or without HTN, or indeed patients with HTN alone. Exaggerated cardiovascular responses to CPT and mental stress have been identified in HTN (Deter et al., 2007, Delaney et al., 2010). A more pronounced increase in HR during CPT was noted in HTN patients. However, RA, RA-HTN and HTN patient groups exhibited similar responses to mental stress, suggesting that the presence of both RA and HTN in an individual does not compound the cardiovascular response to this stressor.

The mechanisms underlying mental stress-induced vasodilation are not fully understood and include: regional sympathetic withdrawal (Halliwill et al., 1997), β-adrenergic mediated vasodilation (Halliwill et al., 1997), flow (shear stress) and nitric oxide mediated vasodilation (Joyner and Tschakovsky, 2003) and circulating factors (e.g. including adrenaline (Lindqvist et al., 1996). Another potential factor is the influence of inflammatory cytokines. Of note, serum IL-10 concentration was positively and independently associated with forearm vasodilatory responses to mental stress. During acute inflammation the release of serum pro-inflammatory cytokines stimulates the production and release of IL-10 (Sabat et al., 2010). Although IL-10 inhibits the synthesis and actions of pro-inflammatory cytokines (TNF-α, IL-1β, IL-6) elevated circulating IL-10 is likely to represent inflammation. This is likely given the strong positive association between IL-10 and other cytokines (TNF-α, IL-6) (Adlan et al., 2017). The release of inflammatory cytokines during acute sepsis is thought to contribute to hypotension via vascular hyporeactivity through a number of suggested mechanisms (Takizawa et al., 1997, Bucher et al., 2001, Bucher et al., 2003, Clapp et al., 2005, Liang et al., 2014). However, while prior studies have shown that TNF-α and IL-1β reduce vascular reactivity to noradrenaline and phenylephrine in animals (Bucher et al., 2003, Liang et al., 2014), to the authors' knowledge no studies have assessed the effects of IL-10 on the vasculature. Future studies are needed to establish the relationship between inflammation and vascular reactivity in healthy humans and disease (including RA).

The cross-sectional design is a limitation of the present study and prevents the establishment of causality between inflammation and HRV, and cardiovascular reactivity. In addition, the use anti-hypertension medication by the RA-HTN and HTN groups was provided according to clinical indication and therefore we cannot exclude the possibility that this influenced HRV and cardiovascular reactivity. We did not ask participants to breathe at a paced rate to ensure that respiratory frequency was in the HF band (i.e., > 0.15 Hz), which could potentially affect the correct interpretation of the frequency domain HRV analyses. However, an estimate of respiratory frequency was derived from the ECG using proprietary software (Kubios) and in the vast majority of participants (57 of 63) it was > 0.15 Hz and only < 0.15 Hz by ~ 0.009 Hz in the others. Importantly, with the latter participants omitted from our analyses there were no major changes to the study findings. We acknowledge the redundancy between RMSSD and SD1 (Brennan et al., 2002). We also acknowledge the relatively small sample size, raising the potential for a type II error, which may contribute to the lack of independent associations in multivariable analyses. Interventional studies using biological agents to inhibit inflammatory pathways are required in RA to confirm whether elevated concentrations of inflammatory cytokines contribute to the autonomic dysfunction reported in such patients. In addition, studies are required to establish the prognostic implications of reduced HRV in RA.

In summary, HRV is reduced in RA and reductions in HRV are independently and inversely associated with reported pain and selected serological markers of inflammation (Ln (hs-CRP)), however HRV was not compounded by the presence of HTN. The existence of autonomic dysfunction in RA (indicated by elevated HR, reduced HRV or cardiac baroreflex sensitivity and increased sympathetic vasoconstrictor activity (Adlan et al., 2017)) likely increases cardiovascular risk, and as such, attempts to control pain and inflammation in RA patients may ameliorate this risk.

## Competing interests

The authors have no conflicts of interest/competing interests.

## Author contributions

JPF, JJCSV, GDK, GYL and JFRP were involved in conception of the work and critical review. AMA and JPF were involved in acquisition, analysis and interpretation of the work. AMA drafted and revised the work. All authors have approved the final manuscript. All authors agree to be accountable for all aspects of the work in ensuring that questions related to the accuracy or integrity of any part of the work are appropriately investigated and resolved. All persons designated as authors qualify for authorship and all those who qualify for authorship are listed.

## Funding

This work was supported by a grant from Arthritis Research UK (grant number 196633).

## Appendix A Supplementary data

Supplementary tables Image 1
