# Supplementary material for: Cardiovascular autonomic regulation, inflammation and pain in rheumatoid arthritis
Source: Auton Neurosci. 2017 Dec;208:137–45. doi: 10.1016/j.autneu.2017.09.003 (PMC5744865; doi:10.1016/j.autneu.2017.09.003)
Supplement: Supplementary file 1 — Supplementary tables [file mmc1.docx]

**SUPPLEMENTARY TABLES**

**S1.** Correlations between pain (VAS), inflammation (hs-CRP, IL-6, TNF-α and IL-10), and heart rate variability.

|  | **Pain VAS** | **hs-CRP** | **IL-6** | **TNF-α** | **IL-10** |
| --- | --- | --- | --- | --- | --- |
| **rMSSD** | **-0.437 ***  **<0.001**  **63** | **-0.420 ***  **0.001**  **57** | **-0.258 ***  **0.043**  **62** | -0.137  0.289  62 | 0.018  0.891  62 |
| **pNN50** | **-0.419 ***  **0.001**  **63** | **-0.430 ***  **0.001**  **57** | -0.226  0.077  62 | -0.074  0.568  62 | 0.040  0.759  62 |
| **LF power** | **-0.367 ***  **0.003**  **63** | **-0.371 ***  **0.004**  **57** | **-0.270 ***  **0.034**  **62** | -0.224  0.080  62 | -0.098  0.451  62 |
| **HF power** | **-0.371 ***  **0.003**  **63** | **-0.348 ***  **0.008**  **57** | -0.205  0.110  62 | -0.138  0.285  62 | -0.098  0.451  62 |
| **LF/HF ratio** | 0.126  0.325  63 | 0.112  0.407  57 | -0.060  0.643  62 | -0.168  0.192  62 | **-0.262 ***  **0.040**  **62** |
| **SD1** | **-0.437 ***  **<0.001**  **63** | **-0.420 ***  **0.001**  **57** | **-0.258 ***  **0.043**  **62** | -0.137  0.289  62 | 0.018  0.891  62 |
| **SD2** | **-0.390 ***  **0.002**  **63** | **-0.344 ***  **0.009**  **57** | **-0.313 ***  **0.013**  **62** | -0.192  0.134  62 | -0.055  0.670  62 |
| **DFA-1** | **0.204**  **0.110**  **63** | **0.171**  **0.203**  **57** | **-0.071**  **0.581**  **62** | -0.148  0.252  62 | -0.251 *  0.049  62 |
| **EDR** | **-0.237**  **0.070**  **59** | **0.048**  **0.718**  **59** | **0.191**  **0.165**  **54** | 0.038  0.777  58 | 0.027  0.841  58 |
| Spearman’s correlation. Values expressed are Spearman’s rho, P value and N. * Significance P≤0.05. | | | | | |

**S2.** Correlations between pain (VAS), inflammation (hs-CRP, IL-6, TNF-α and IL-10), and cardiovascular responses to the PASAT and CPT.

|  | **Pain VAS** | **hs-CRP** | **IL-6** | **TNF-α** | **IL-10** |
| --- | --- | --- | --- | --- | --- |
| **Systolic BP response to PASAT** | -0.120  0.408  50 | 0.018  0.905  46 | **-0.287 ***  **0.044**  **50** | -0.107  0.461  50 | -0.141  0.328  50 |
| **Diastolic BP response to PASAT** | -0.120  0.408  50 | 0.017  0.912  46 | -0.177  0.220  50 | -0.056  0.699  50 | -0.145  0.315  50 |
| **Mean BP response to PASAT** | -0.084  0.561  50 | 0.010  0.949  46 | -0.151  0.296  50 | -0.017  0.909  50 | -0.088  0.541  50 |
| **HR response to PASAT** | -0.244  0.088  50 | -0.195  0.193  46 | -0.133  0.355  50 | -0.009  0.950  50 | 0.187  0.194  50 |
| **Leg blood flow response to PASAT** | 0.171  0.240  49 | 0.287  0.056  45 | 0.161  0.268  49 | 0.104  0.475  49 | 0.061  0.678  49 |
| **LVC response to PASAT** | 0.179  0.218  49 | 0.246  0.103  45 | 0.237  0.101  49 | 0.159  0.274  49 | 0.121  0.406  50 |
| **Forearm blood flow response to PASAT** | -0.113  0.435  50 | 0.035  0.816  46 | 0.081  0.577  50 | 0.148  0.305  50 | 0.198  0.168  50 |
| **FVC response to PASAT** | -0.124  0.392  50 | 0.002  0.991  46 | 0.104  0.473  50 | 0.160  0.267  50 | 0.250  0.080  50 |
| **Systolic BP response to CPT** | -0.057  0.664  60 | -0.036  0.793  55 | 0.081  0.536  60 | 0.185  0.156  60 | 0.112  0.395  60 |
| **Diastolic BP response to CPT** | -0.103  0.432  60 | -0.023  0.865  55 | 0.143  0.277  60 | 0.245  0.059  60 | 0.212  0.103  60 |
| **Mean BP response to CPT** | -0.093  0.479  60 | 0.017  0.900  55 | 0.139  0.289  60 | 0.232  0.074  53 | 0.179  0.171  60 |
| **HR response to CPT** | -0.100  0.447  60 | -0.147  0.285  55 | 0.113  0.388  60 | **0.254 ***  **0.050**  **60** | **0.299 ***  **0.020**  **60** |
| **Leg blood flow response to CPT** | -0.202  0.129  58 | -0.022  0.874  55 | -0.086  0.520  60 | -0.0574  0.670  58 | -0.118  0.380  58 |
| **LVC response to CPT** | -0.100  0.447  60 | -0.086  0.543  55 | -0.082  0.543  60 | -0.040  0.765  58 | -0.142  0.287  58 |
| Spearman’s correlation. Values expressed are Spearman’s rho, P value and N. * Significance P≤0.05. | | | | | |
